# A genome-wide scan to identify loci for smoking rate in the Framingham Heart Study population

**DOI:** 10.1186/1471-2156-4-S1-S103

**Published:** 2003-12-31

**Authors:** Ming D Li, Jennie Z Ma, Rong Cheng, Randolph T Dupont, Nancy J Williams, Karen M Crews, Thomas J Payne, Robert C Elston

**Affiliations:** 1 Department of Psychiatry, The University of Texas Health Science Center at San Antonio, San Antonio, Texas, USA; 2 Departments of Psychiatry and Allied Health, The University of Tennessee Health Science Center, Memphis, Tennessee, USA; 3 Departments of Diagnostic Sciences and Medicine, The University of Mississippi Medical Center, Jackson, Mississippi, USA; 4 Department of Epidemiology and Biostatistics, Case Western Reserve University, MetroHealth Drive, Cleveland, Ohio, USA

## Abstract

**Background:**

Although many years of genetic epidemiological studies have demonstrated that genetics plays a significant role in determining smoking behavior, little information is available on genomic loci or genes affecting nicotine dependence. Several susceptibility chromosomal regions for nicotine dependence have been reported, but few have received independent confirmation. To identify susceptibility loci for nicotine dependence, 313 extended pedigrees selected from the Framingham Heart Study population were analyzed by both the GENEHUNTER and S.A.G.E. programs.

**Results:**

After performing linkage analyses on the 313 extended Framingham Heart Study families, the EM Haseman-Elston method implemented in GENEHUNTER provided evidence for significant linkage of smoking rate to chromosome 11 and suggestive linkage to chromosomes 9, 14, and 17. Multipoint sib-pair regression analysis using the SIBPAL program of S.A.G.E. on 1389 sib pairs that were split from the 313 extended families identified suggestive linkage of smoking rate to chromosomes 4, 7, and 17. Of these identified positive regions for nicotine dependence, loci on chromosomes 7, 11, and 17 were identified by both GENEHUNTER and S.A.G.E. programs.

**Conclusion:**

Our genome-wide scan results on the Framingham Heart Study data provide evidence for significant linkage of smoking rate to chromosome 11 and suggestive linkage to chromosomes 4, 7, 9, 14, and 17. These findings suggest that some of these regions may harbor susceptibility loci for nicotine dependence, and warrant further investigation in this and other populations.

## Background

Over the last several decades, a number of twin studies throughout the world have yielded results consistent with the overall conclusion that both genetic and environmental factors contribute to the risk of becoming a long-term smoker (for reviews, see [1,2]). After performing a meta-analysis of most of the reported twin studies on smoking-related behaviors in the literature, we found that genetic factors contribute approximately 50% to smoking initiation and 59% to smoking persistence [3].

Although the twin studies suggest moderate genetic influences on nicotine dependence, little information is provided about the chromosomal locations harboring susceptibility loci/genes for nicotine dependence. Linkage-based genome-wide scans for smoking behavior have been reported by Straub et al. [4] on the Christchurch sample of New Zealand (130 families with 343 genotyped individuals) and the Richmond sample of Virginia (91 families with 264 genotyped individuals), and by Duggirala et al. [5] and Bergen et al. [6] on the Collaborative Study on the Genetics of Alcoholism data (COGA; 105 families with 987 genotyped individuals). However, only a few susceptibility regions for nicotine dependence from one study were replicated in another study.

Generally speaking, there are two approaches available to address this problem of identifying susceptibility loci for nicotine dependence and other complex disorders. The first approach is to repeat and extend these genome-wide linkage analyses in different populations; the second is to use higher marker densities for association genome scanning studies. Based on the availability of information on smoking phenotype in the Framingham Heart Study population, we adopted the first genome-wide scan approach to identify susceptibility loci for nicotine dependence in the present study.

## Methods

Data from the Framingham Heart Study along with clinical exam information from 1948 through 1988 for the original cohort and from 1971 through 1991 for the offspring cohort were provided through Genetics Analysis Workshop 13 (GAW13). On the basis of number of smokers present at each exam, the consistency of the clinical data and interviewing time between the two cohorts, and the potential environmental effect on smoking phenotype included in the Framingham Heart Study data, Exam 12 from 1970 for the original cohort and Exam 1 from 1971 for the offspring cohort were selected and used in this study.

From the 330 extended families of the Framingham Heart Study, 313 were chosen in which there was at least one smoker present in the data from 1970–1971. Table 1 shows some of the characteristics of the sample used in the current study. The smoking rate (SR) phenotype of each individual reported in the study was based on self-reported average number of cigarettes smoked per day during 1970–1971. Information on smoking rate was available for 2493 of the 4522 members distributed across the 313 families. Of these 2493 subjects, 1636 were genotyped for 401 markers at an average spacing of 7.5 cM between markers. For individuals who reported non-smoking, we considered their phenotype as 'zero' and included them in the linkage analysis. On the other hand, we considered the phenotypes of individuals who did not report their smoking status during the survey as 'unknown' and excluded them from the analysis. Skewness and kurtosis for the self-reported average number of cigarettes smoked per day were 1.38 and 5.11, respectively. To minimize the impact of skewness on linkage analysis results, we transformed the non-zero smoking rates to a natural log-scale prior to linkage analysis (called log-transformed SR). Skewness and kurtosis for this log-transformed smoking rate (including the zeros) became 0.31 and 1.30, respectively. Additionally, we generated a third data set for smoking rate (called categorized SR) in which individuals who smoked 0, 1–5, 6–15, 16–25, 26–35, and greater than 35 cigarettes per day were assigned the values 0, 1, 2, 3, 4, and 5, respectively. Skewness and kurtosis for the categorized SR were 0.58 and 1.88, respectively.

Two linkage analysis programs (SIBPAL in S.A.G.E. v. 4.2 and GENEHUNTER v. 2.1) were used in the study. For the EM (expectation maximization) Haseman-Elston quantitative trait locus (QTL) regression method implemented in GENEHUNTER, we analyzed both log-transformed and categorized SR data sets. A detail description on the method can be found in Kruglyak et al. [7] and Kruglyak and Lander [8]. In SIBPAL, default options were used for all parameters in the trait regression method except that the options w3 (the weighted combination of squared trait difference and squared mean-corrected trait sum adjusting for the non-independence of sib pairs) and w4 (the non-independence of squared trait sums and differences) were examined [9]. Both options yielded essentially the same results on three data sets (i.e., SR, log-transformed SR and categorized SR). Sex and age were included as covariates for all analyses reported in this communication. The S-PLUS 6.1 and SAS 8.2 packages were used to prepare the data in the required format and to analyze the data generated from the linkage analysis programs.

## Results

To maximally utilize the phenotypic information from the Framingham Heart Study data, we searched clinical records regarding smoking status of each subject from 1948 to 1988 for the original cohort, and from 1971 to 1989 for the offspring cohort. It appears that data from 1970 for the original cohort and from 1971 for the offspring cohort were more complete and contained significantly more smokers relative to other time points (i.e., exams) for both cohorts. As shown in Table 1, 1228 smokers were included in the study, with an average daily smoking rate of 23.51 and 17.95 cigarettes for men and women, respectively. The average age of smokers included in this study was 38.7 ± 13.4 for male and 39.73 ± 13.8 for female subjects.

After performing linkage analysis on both log-transformed and categorized smoking rate data sets with the EM Haseman-Elston QTL regression method implemented in GENEHUNTER, we found evidence for significant linkage of smoking rate to chromosome 11 in both the log-transformed (*p *= 0.000018) and the categorized SR data sets (*p *= 0.000001), based on the threshold suggested by Nyholt [10]. We also found evidence for suggestive linkage of smoking rate to chromosomes 9, 14, and 17 (*p *< 0.0017; [10]). Further, we found several regions located on chromosomes 7, 15, and 20 to be of potential interest at a significance level of *p *< 0.01 (Table 2). To confirm these findings, we analyzed the original and transformed SR data sets using the SIBPAL program in S.A.G.E. (v. 4.2) with the option of splitting these extended families into nuclear families, which yielded 1389 sib pairs. Three loci for which SIBPAL provided evidence for suggestive linkage were mapped onto chromosomes 4, 7, and 17. In addition, we found six more loci of potential interest at a significant level of 0.01 (Table 3).

## Discussion

In this study, by using the EM Haseman-Elston QTL regression method implemented in GENEHUNTER and the SIBPAL program of S.A.G.E., we obtained evidence for significant linkage of smoking rate to chromosome 11 and suggestive linkage to chromosomes 4, 7, 9, 14, and 17. Additionally, our results suggest that the genomic regions mapped on chromosomes 1, 6, 12, 15, 20, and 21 are of potential interest to harbor susceptibility genes for nicotine dependence at a significance level of 0.01. Of these loci, three loci on chromosomes 7, 11, and 17 were identified by both linkage analysis methods.

Although our mapping results provided weak evidence for linkage of smoking rate to chromosome 20 (*p *= 0.0063; see Table 2), this locus appears to be interesting. Using the same Framingham Heart Study data, two other research groups [11,12] independently reported weak linkage of the maximum number of cigarettes smoked per day across the first four exams or across all exams of the original and offspring cohorts onto the same region of chromosome 20 identified in this study. To our knowledge, no previous studies in the literature have identified linkage or association with this region on chromosome 20. Therefore, it will be of interest to confirm this finding in other studies. The reason that we achieved lower *p-*values for most regions reported in our study than other GAW13 analyses evaluating smoking as the phenotype may due to how the data measuring smoking phenotype were selected from the Framingham Heart Study data. As indicated earlier, we only used the smoking information obtained from the exam conducted during 1970–1971, instead of using the maximum number of cigarettes smoked per day across multiple exams over many years. Epidemiological studies have indicated that there has been a steady and dramatic decline of 40% in the prevalence of cigarette smoking by people 18 years or older in the US from 1965 to 1990 [13]. This was also true in the Framingham Heart Study data (data not shown). Therefore, using smoking information obtained from multiple exams over a long period of time may affect estimation of the genetic and environmental parameters, and thus eventually the linkage analysis results. Nicotine dependence is a complex trait with strong genetic and environmental influences. Many years of genetic epidemiological studies have documented that smoking behavior is determined by multiple genetic and environmental factors, and interaction among these factors. Strong evidence for linkage of smoking behavior to chromosome 5q has been reported from an analysis of the COGA data [5]. The linkage to smoking behavior on chromosome 5 was also reported by another study with a different linkage analysis method but at a marginal level of significance [6]. In another independent study, Straub et al. [4] identified several possible regions for nicotine dependence on chromosomes 2, 4, 10, 16, 17 and 18 in the Christchurch sample of New Zealand but failed to confirm these regions in the Richmond sample of Virginia. This was probably due to insufficient statistical power as a result of the small sample size of the Richmond cohort (91 families with 264 genotyped individuals). Compared with the research described above, a much larger sample size was used in the present study, which may contribute partially to the significant *p*-values obtained in this study.

There are limitations to this study. For example, we used the number of cigarettes smoked per day as an indirect measure of nicotine dependence without consideration of which cigarette brand each smoker smoked. It is known that there exists a significant variation in nicotine concentration present in each cigarette brand. Therefore, the phenotype of smoking rate used here may represent only a very rough measure of nicotine dependence. Given the objective of this study and the limitation of the data set used in the analysis, we did not, nor were we able to, distinguish individuals in the non-smoking group who had been passively exposed to smoking (i.e., through second-hand smoke) from those who were never exposed to nicotine. As documented earlier, the transformed smoking phenotype still deviated slightly from a normal distribution; however, we do not feel that such remaining kurtosis would have a large effect on the linkage results reported herein, because only model-free methods were used in the analysis and they tend to be more robust to the presence of non-normality in the data. Also, the participants in the Framingham Heart Study are predominantly Caucasian Americans. Accordingly, it is of interest to know whether we can repeat these findings in other ethnic populations.

## Conclusions

The 313 extended Framingham Heart Study families were analyzed to identify susceptibility loci for smoking rate by the Haseman-Elston regression methods implemented in GENEHUNTER and the SIBPAL program of S.A.G.E. Our genome-wide scan results provided evidence for significant linkage of smoking rate to chromosome 11 and suggestive linkage to chromosomes 4, 7, 9, 14, and 17. Additionally, we found several regions located on chromosomes 1, 6, 12, 15, 20, and 21 are potentially of interest with a significance level of <0.01. Interestingly, the genomic regions on chromosomes 7, 11, and 17 were identified by both the linkage methods. To our knowledge, most of the susceptibility regions for smoking rate identified in this study have not been reported previously and thus replication of these findings is an important next step.

## Figures and Tables

**Table 1:** Characteristics of pedigrees to map susceptibility loci for smoking rate.

**Category**	**Mean ± SEM (Range)**	**Sample Size**^A^
No. of subjects per pedigree	8.25 ± 0.31 (3–53)	313
No. of smokers per pedigree	3.92 ± 0.16 (1–25)	313
Age of subjects	43.76 ± .34 (9–83)	2583
Male subjects	42.92 ± 0.49 (11–82)	1242
Female subjects	44.54 ± 0.46 (9–83)	1341
Age of smokers	39.23 ± 0.39 (13–80)	1228
Male smokers	38.73 ± 0.54 (15–79)	614
Female smokers	39.73 ± 0.56 (13–80)	614
Self-reported cigarettes per day, 1970–1971	20.73 ± 0.35 (1–90)	1228
Male smokers	23.51 ± 0.53 (1–90)	614
Female smokers	17.95 ± 0.43 (1–60)	614

^A^The unit of measurement is number of pedigrees for the first two and number of individual subjects for all other rows.

**Table 2:** The *p*-value for genomic locations linked to categorized and log-transformed SR across the human genome with a *p*-value < 0.01 for at least one transformed data set, as detected by the EM-Haseman-Elston method in GENEHUNTER.

**Chromosome**	**Locus**	**Log-transformed SR (peak position)**	**Categorized SR (peak position)**
7	GATA31A10 & GATA24D12	0.0087 (64)	>0.05 (64)
9	183xh10 & ATA18A07	0.0039 (106)	0.00057^B ^(106)
11	GGAA5C04 & GATA90D07	0.000018^A ^(70)	0.000001^A ^(70)
14	GATA51E09 & GGAA21G11	0.0011^B ^(111)	>0.05 (111)
15	GATA73F01 & GATA27A03	0.0037 (127)	0.0247 (127)
17	ATA78D02 & GATA185H04	0.0043 (37)	0.00075^B ^(41)
20	GATA47F05 & 321xd1	0.0139 (82)	0.0063 (82)

^A^Significant linkage and ^B ^Suggestive linkage according to the threshold suggested by Nyholt [10].

**Table 3:** The *p*-value for genomic locations linked to smoking rate (SR), categorized and log-transformed SR across the human genome with a *p*-value for at least one transformed data set, as detected by SIBPAL program.

**Chromosome**	**Locus**	**SR (peak position)**	**Log-transformed SR (peak position)**	**Categorized SR (peak position)**
1	GGAA5F09	>0.05 (188)	0.0045 (188)	0.0347 (188)
4	GATA42H02 & 165xc11	0.0017^A ^(217)	0.0042 (203)	0.0036 (205)
6	SE30	0.0030 (10)	>0.05 (10)	>0.05 (10)
7	GATA31A10	>0.05 (63)	0.0016^A ^(63)	0.0044 (63)
11	GGAA5C04 & GATA90D07	>0.05 (68)	0.0084 (68)	0.0097 (72)
12	GATA3F02	0.0021 (88)	>0.05 (88)	>0.05 (88)
17	ATA78D02 & GATA185H04	0.0004^A ^(45)	0.0106 (45)	0.0225 (45)
17	ATC6A06 & GATA49C09	0.0095 (87)	>0.05 (87)	>0.05 (87)
21	GATA188F04	0.0096 (44)	>0.05 (44)	>0.05 (44)

^A^Suggestive linkage according to the threshold suggested by Nyholt [10].
